# Ethical perspectives on surgical video recording for patients, surgeons and society: systematic review

**DOI:** 10.1093/bjsopen/zrad063

**Published:** 2023-06-24

**Authors:** Ross Walsh, Emma C Kearns, Alice Moynihan, Sara Gerke, Mindy Duffourc, Marcelo Corrales Compagnucci, Timo Minssen, Ronan A Cahill

**Affiliations:** Department of Surgery, Mater Misericordiae University Hospital, Dublin, Ireland; UCD Centre of Precision Surgery, University College Dublin, Dublin, Ireland; UCD Centre of Precision Surgery, University College Dublin, Dublin, Ireland; PennState Dickinson Law, Pennsylvania State University, Carlisle, Pennsylvania, USA; PennState Dickinson Law, Pennsylvania State University, Carlisle, Pennsylvania, USA; New York University School of Law, New York University, New York, New York, USA; Centre for Advanced Studies in Biomedical Innovation Law (CeBIL), University of Copenhagen, Copenhagen, Denmark; Centre for Advanced Studies in Biomedical Innovation Law (CeBIL), University of Copenhagen, Copenhagen, Denmark; Department of Surgery, Mater Misericordiae University Hospital, Dublin, Ireland; UCD Centre of Precision Surgery, University College Dublin, Dublin, Ireland

## Abstract

**Background:**

Operating-room audiovisual recording is increasingly proposed, although its ethical implications need elucidation. The aim of this systematic review was to examine the published literature on ethical aspects regarding operating-room recording.

**Methods:**

MEDLINE (via PubMed), Embase, and Cochrane databases were systematically searched for articles describing ethical aspects regarding surgical (both intracorporeal and operating room) recording from database inception to the present (the last search was undertaken in July 2022). Medical subject headings used in the search included ‘operating room’, ‘surgery’, ‘video recording’, ‘black box’, ‘ethics’, ‘consent’, ‘confidentiality’, ‘privacy’, and more. Title, abstract, and full-text screening determined relevance. The quality of studies was assessed using Centre for Evidence-Based Medicine grading and no formal assessment of risk of bias was attempted given the theoretical nature of the data collected.

**Results:**

From 1048 citations, 22 publications met the inclusion criteria, with three more added from their references. There was evident geographical (21 were from North America/Europe) and recency (all published since 2010) bias and an exclusive patient/clinician perspective (25 of 25). The varied methodology (including ten descriptive reviews, seven opinion pieces, five surveys, two case reports, and one RCT) and evidence level (14 level V and 10 level III/IV) prevented meaningful systematic grading/meta-analysis. Publications were narratively analysed for ethical thematic content (mainly education, performance, privacy, consent, and ownership) that was then grouped by the four principles of biomedical ethics of Beauchamp and Childress, accounting for 63 distinct considerations concerning beneficence (22 of 63; 35 per cent), non-maleficence (17 of 63; 27 per cent), justice (14 of 63; 22 per cent), and autonomy (10 of 63; 16 per cent). From this, a set of proposed guidelines on the use of operative data is presented.

**Conclusion:**

For a surgical video to be a truly valuable resource, its potential benefits must be more fully weighed against its potential disadvantages, so that any derived instruments have a solid ethical foundation. Universal, ethical, best-practice guidelines are needed to protect clinicians, patients, and society.

## Introduction

The concept of surgery as a spectacle is not new. There is a long and continuing tradition of operative performance being independently viewed^1^. Indeed, since the 19th century^2^, surgeons have performed operations specifically in rooms called ‘theatres’, initially in front of crowds either standing or in tiered seating. More recently, as modern surgery developed aseptic techniques and environments, as well as defined training curricula^3^, operating rooms (OR) often include viewing areas and screens for trainees and other observers to watch. With the broad move towards minimally invasive surgery with digital cameras and the capability for surgical video display, transmission, and recording, large audiences are now being reintroduced to surgery. Surgeries can now be watched back for surgical (including patient and public) education, training, and development^4^. In conjunction with artificial intelligence (AI) models, surgical video recordings enable the creation of OR black boxes (ORBB)^5,6^ that have the potential to further transform the field by providing richer detail than traditional operative notes, with AI promising automatic postoperative analysis and insights and potentially intraoperative decision support^7,8^. Thus, surgical videos now have more interest and value than ever before.

Yet, there are concerns over the ethical and legal implications of surgical video recording and the resulting data processing, both in terms of its sourcing and use. Some legal aspects have been discussed elsewhere^9–14^, and both the European General Data Protection Regulation (GDPR)^15^ and US Health Insurance Portability and Accountability Act (HIPAA)^16^ provide legal frameworks to protect citizen privacy, but ethical considerations have been less formally addressed (although of course ethics and law are often intertwined, such as with regard to privacy and ownership issues). There are methods, however, such as the framework of the principles of biomedical ethics of Beauchamp and Childress^17^ (namely respect for beneficence, non-maleficence, autonomy, and justice^8–11,18^), which can help to identify, address, and ultimately solve ethical medical dilemmas.

The aim of this systematic review was to examine the published literature on ethical aspects of OR data collected via video and/or audio recording, using the principles of Beauchamp and Childress to categorize perspectives, and consider their consequences for individuals (patients and medical staff) and also the general population. Like all systematic reviews, its purpose was to draw together and analyse the existing evidence base, to identify current best practice and gaps that need to be addressed in the future.

## Methods

This systematic review (registered with the international prospective register of systematic reviews PROSPERO^19^—CRD42022348406) was completed in accordance with the PRISMA^20^ guidelines.

### Study objective

To examine the published English-language literature on ethical aspects in relation to OR data collected via video and/or audio recording, as well as their implications for individual patients and medical staff, as well as the general population.

### Search strategy

MEDLINE (via PubMed), Embase, and Cochrane databases were searched from database inception to the present (the last search was undertaken in July 2022). There were no limits or restrictions on the basis of date or language of publication at the time of searching; however, only articles available in English were included in the full-text review. Medical subject headings used in the search included ‘operating room’, ‘surgery’, ‘video recording’, ‘black box’, ‘ethics’, ‘consent’, ‘confidentiality’, ‘privacy’, and more (see *Table S1* for the complete search strategy). The references of included publications were also searched to ensure completeness.

### Eligibility criteria

Surgical audiovisual recording was defined as any recording from intracorporeal recording devices (procedural video), as well as medical device recorders recording the OR and/or the procedure itself (panoramic video), including ORBB^21^ and Google Glass^22^ technology. Although live surgery broadcasting^13,23–30^ and tele-surgery^31,32^ were beyond the scope of the specific focus here, they are also relevant areas for similar elucidation. The implications of video recording outside the OR, for example in endoscopy suites and critical care areas, were also excluded. Full inclusion and exclusion criteria are outlined in *Table 1*. In brief, publications were eligible for inclusion if they included ‘ethics’ or an ethical aspect (including, but not limited to, benefits, privacy, confidentiality, ownership, and consent) in the title or abstract in relation to surgical videos and data and medical device recorders. Original research (randomized controlled, observational, cohort, case–control, case series, and cross-sectional) studies, as well as reviews and commentaries, published in peer-reviewed journals were included.

**Table 1:** Inclusion/exclusion criteria

Inclusion criteria	Exclusion criteria
Include ethics or ethical aspect in title/abstract with regards to surgical videos, medical device recorders, or surgical data	Publications that discussed OR recording, but made no reference to ethics/ethical principle
Contemporary peer-reviewed literature, including RCTs, cohort publications, case–control publications, case series, review articles, and commentary articles	Video recording outside of the OR
Any publication date, published in English language	Non-English publication
If multiple articles had overlapping cohorts (determined by institution and year), only the most recent publication was included	Live surgery
	Tele-surgery

OR, operating room.

### Study selection

After the removal of duplicates, titles and abstracts were screened for relevance by two reviewers (R.W. and E.C.K.). R.W. and E.C.K. then reviewed the full-text articles according to the inclusion and exclusion criteria as outlined in *Table 1*. The text and reference lists of included articles were manually searched for additional articles of interest, which were assessed according to the same outlined inclusion and exclusion criteria. Discrepancies that occurred at the title- and abstract-screening stages were resolved by automatic inclusion. Discrepancies at the full-text stage were resolved by consensus between the two reviewers and, if disagreement persisted, a third reviewer (R.A.C.) was consulted.

### Data extraction

Relevant data were collected by a single reviewer (R.W.) using a predefined pro forma. Data were sought on the following items from each included article: study characteristics (title, first author, country of origin, and year of publication), study design, and ethical aspect. The quality of studies was assessed using Centre for Evidence-Based Medicine (CEBM)^33^ grading and assessment followed the guidelines of the National Centre for Research Methods on conducting narrative synthesis^34,35^. A publication’s perspectives were grouped thematically under one or more of the four principles of biomedical ethics of Beauchamp and Childress: beneficence, non-maleficence, autonomy, and justice^8–11^. The total number of times each ethical principle was discussed was tallied, taking into account the fact that multiple principles could be discussed in each paper, and the data presented systematically. No formal assessment of risk of bias was attempted given the theoretical nature of the data collected.

## Results

### Search results

**Fig. 1:**
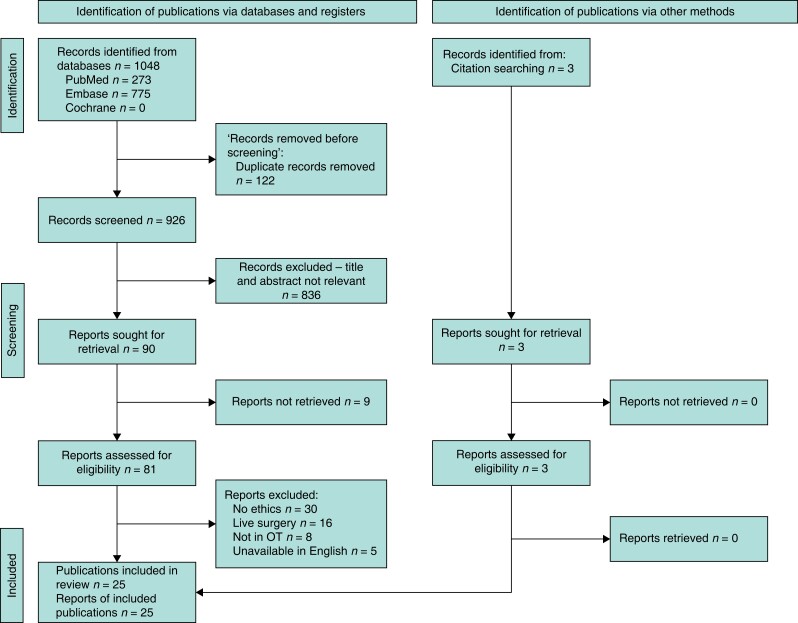
**PRISMA flow diagram** Permission granted. Creative Commons Attribution License, which permits unrestricted use. OT, operating theatre.

A PRISMA flow diagram of the study selection process is presented in *Fig. 1*. Following the search strategy, 1048 potentially eligible publications were identified. After removing duplicates, unrelated fields, abstracts without full text, and non-relevant papers, 22 manuscripts met the inclusion criteria, with a further 3 publications being added from their references.

**Fig. 2:**
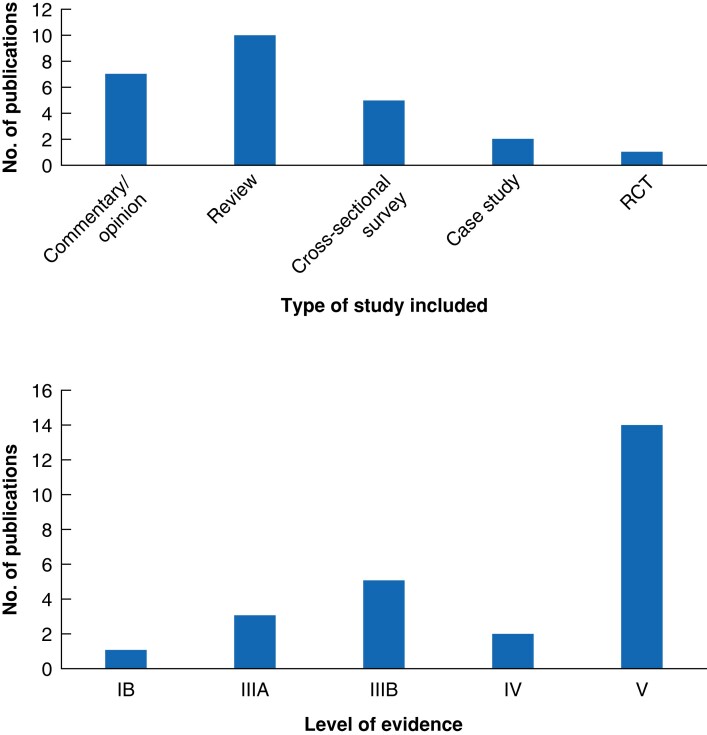
**Quality of evidence regarding study types included as per the Centre for Evidence-Based Medicine** ^33^ A total of 25 publications were included.

Publications were primarily from North America (52 per cent) and Europe (32 per cent), but also came from Australia, Asia, and South America. Publication types included commentaries and surveys/interviews, as well as descriptive, opinion, and narrative reviews (see *Table 2*) of greatly varying CEBM quality (see *Fig. 2*). Given this diversity, it was not possible to formally grade the evidence or to undertake statistical analysis. Included publications were therefore synthesized narratively.

**Table 2:** Summary of included publications, including year of publication, first author, country of origin, study type, modality type, and ethical themes explored

Year	First author	Location	Type of study	Type of modality	Ethical themes explored
2022	Jesudason^36^	UK	Commentary/opinion	Video recording	Transparency, AI to improve surgery, confidentiality, consent, cost, fairness, distribution
2022	Gordon^37^	Canada	Cross-sectional survey	MDR: ORBB	Confidentiality
2022	Filicori^38^	USA	Review/survey	Video recording	Confidentiality, security, consent, ownership, fairness
2022	Cahill^39^	Ireland	Commentary/opinion	Video recording	Performance, AI to improve surgery, quality improvement, editing, ownership
2021	Gallant^40^	USA	Cross-sectional interview	MDR	Education, performance, transparency, confidentiality, consent, editing, ownership, cost, fairness
2020	Jue^41^	USA	Systematic review	MDR	Performance, quality improvement
2020	Gabrielli^42^	Chile	Narrative/literature review	MDR	Education, performance, quality improvement, confidentiality, open discussion, consent, editing, ownership
2020	Darrow^43^	USA	Cross-sectional survey	MDR: ORBB	Education, confidentiality, distribution
2020	Doyen^21^	Belgium	Case study	Video recording	Education, quality improvement, fairness
2019	van Dalen^11^	Netherlands	Systematic review	MDR	Confidentiality, consent, ownership
2019	Thia^44^	Australia	Literature review	Video recording	Education, performance, transparency, AI to improve surgery, quality improvement, confidentiality, security, consent, editing, ownership
2018	Hung^45^	USA	Systematic review	Video recording	Education
2017	Langerman^46^	USA	Commentary/opinion	MDR, video recording	Education, performance, transparency, quality improvement, open discussion
2016	Prigoff^47^	USA	Commentary/opinion	Video recording	Performance, transparency, confidentiality, security, consent, editing, ownership
2016	Chang^22^	Hong Kong	Literature review	Video recording: Google Glass	Education, performance, confidentiality
2016	O'Mahoney^48^	USA	Commentary/opinion	Video recording	Performance, transparency, quality improvement, confidentiality
2016	Evans^32^	USA	Literature review	Video recording	Education
2016	Grenda^49^	USA	Commentary/opinion	Video recording	Performance
2015	Bonrath^50^	Canada	Randomized controlled trial	Video recording	Performance, quality improvement
2015	Silas^51^	USA	Cross-sectional survey	Video recording	Confidentiality
2014	Turnbull^52^	UK	Literature review	Video recording	Education, performance, confidentiality, open discussion, security, consent, editing, ownership, distribution
2013	Couat^53^	France	Case study	MDR	Quality improvement
2012	Henken^54^	Netherlands	Literature review	Video recording	Confidentiality, ownership
2007	Xiao^55^	USA	Commentary/opinion	Video recording	Education, performance, quality improvement, confidentiality, consent, ownership
2007	Kocyildirim^56^	UK	Cross-sectional survey	Video recording	Transparency, ownership

AI, artificial intelligence; MDR, medical device recording; ORBB, operating room black box.

### Narrative synthesis

**Fig. 3:**
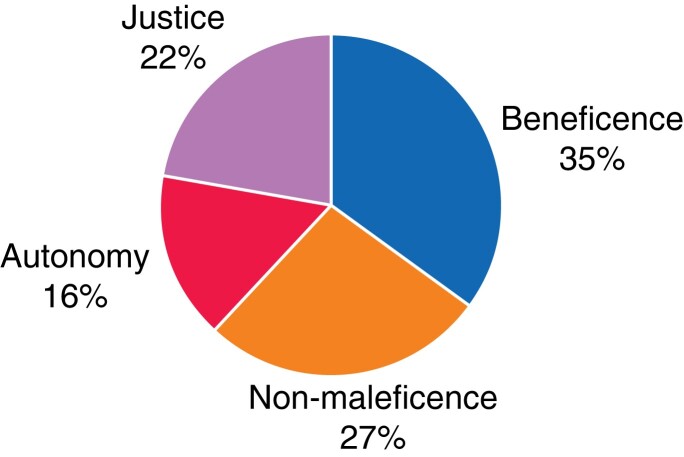
Ethical themes covered in the 25 included papers There were 63 distinct discussions based on one of the four principles of biomedical ethics, with the breakdown between beneficence, non-maleficence, justice, and autonomy being shown in the figure.

*Table 3* summarizes the areas of strongest evidence for the use of operative data, which can provide a basic guidance of sorts from this review^34^. In total, from the 25 papers, there were 63 distinct discussions of the principles of Beauchamp and Childress (often publications discussed more than one principle; see *Table S2* for more detail). Beneficence was discussed in 22 of 25 publications, comprising 35 per cent of total discussion detail, with non-maleficence, justice, and autonomy being discussed in 17 (27 per cent), 14 (22 per cent), and ten (16 per cent) papers respectively (see *Fig. 3*). Considerations over confidentiality (15 publications), ownership (11 publications), and consent (9 publications) were also raised frequently.

**Table 3:** Proposed guidelines on operative data

Creation of a video/audio recording should have a clearly stated purpose. This may include educational, research, quality improvement, patient request, or others.
Any patient undergoing a procedure that may include recording should be made aware and properly consented. This includes, but is not limited to, the purpose of the recording, the intended audience, and the parts of the procedure recorded. Consent should be able to be withdrawn at any stage.
Data should be encrypted and ideally anonymized (although pseudonymized may be needed for certain purposes where access to clinical data via a key may be justified) and stored on secure platforms or servers.
Patients, faculty, and staff should be notified that a recording will take place during the procedure and given the opportunity to opt out.
If editing is required for visual accuracy or timeliness for a presentation, the alterations should be clearly disclosed to any audience.
Data ownership/access rights should be clear at inception and contact details given for future enquiries.
All recordings should be protected with the same security and scrutiny that the hospital and physicians use for patients’ medical records.

### Beneficence

Benefits discussed included performance (13 publications), education and research (11 publications), transparency and improved patient understanding (7 publications), quality improvement and safety (10 publications), and field advancement through AI (3 publications).

#### Performance

Positive change in performance was attributed, in general, to *post-hoc* case review, improvement in technical skill, and the Hawthorne effect. Eight publications discussed the learning and reflective opportunity from re-watching operations^49^. Six discussed how such analysis can improve operative skill (including improved error detection) and one review discussed the use of motion-analysis software and AI to examine and aid improvement^44^. Six also discussed the Hawthorne effect (also known as the ‘observer effect’), which is defined as a change in normal behaviour when individuals are aware they are being observed. Four publications^42,46,52^ acknowledged that this could have a positive or negative effect, with improvement being possibly attributed to increased accountability, attentiveness, and meticulousness, whereas a negative effect could be due to anxiety, stress, or theatrics. Enhanced performance due to improved intraoperative communication was also discussed^22,55^, as was the use of the recording to optimize OR dynamics^46^.

#### Education/research

All publications concerning education/research advocated that operative recording could benefit surgical trainees either generally or more specifically through video-based coaching and tele-mentoring^42,44^, intraoperative engagement^22^, and targeted feedback^21^. Two articles discussed the benefits of recording for all theatre staff in learning OR dynamics^42,55^.

#### Transparency/patient understanding

Seven articles discussed transparency and increased patient understanding as a key benefit of operative data recording^36,40,44,46–48,56^. Six proposed that such recording provides a clear objective record of what happened during surgery instead of relying on memory and self-reported dictation/notes, thus reducing bias in the OR by ensuring that there is a record of all operative steps that might otherwise be missed^57^. Four of the seven articles discussed improved patient understanding, which can lead to more informed health decisions^46^ and even help patients come to terms with the emotional trauma of surgery^46^. Recordings may also act as an aid to open disclosure and duty of candour when explaining complications to patients^58^.

#### Quality improvement/safety

Eight publications specified quality-improvement usefulness through the detection of OR errors, with two publications attributing this to improved documentation and audit^21,39,41,42,44,46,48,50,53,54^. Operative recording allows the types of errors assessed to be broken down into technical (that is surgical steps; discussed in seven publications^21,41,42,48,50,53,55^) and non-technical (relating to the OR environment, including OR dynamics, human and system factors, radiation safety, and prevalence of distractions; discussed in four publications^21,42,46,53^) errors, either alone (three publications for technical errors and one publication for non-technical errors) or in combination.

#### Artificial intelligence to further surgery

One author group discussed how AI can be used to look for patterns associated with success and failure, to identify better surgeons^36^, with another stating that AI access to outcome-linked operative videos could allow for quality improvement, allow for new technique and technology development, and give us a better insight into surgery itself^39^. A further review delineated the use of motion-analysis software and AI pattern recognition, already in use in ophthalmic surgery^44^.

### Non-maleficence

Concerns discussed related to privacy and confidentiality (15 publications; 5 publications discussed privacy alone), storage and security (5 publications), and open discussion (3 publications).

#### Privacy/confidentiality

Patient confidentiality was the main area of discussion^22,36–38,40,42–44,47,48,51,52,54,55,59^ (15 publications) with reference to European and US privacy laws. A cross-sectional survey of operating-theatre staff showed that 45 per cent of respondents were concerned about OR recording data security. The personal privacy of both patients and OR staff themselves was considered in five publications. One study involving in-theatre recording using a Google Glass headset quantified this risk to patients as 0.4 exposures or potential privacy breaches per minute. Exposures included patients’ faces or any identifying information being accidentally recorded, with such an exposure occurring every 2.5 min of operating time^43^. Two publications offered data encryption^38^ and anonymization^51^ to mitigate these concerns.

#### Preventing open discussion

Three publications discussed the potential harms of OR recordings and their consequences and concerns regarding education and surgical performance^42,46,52^. OR recording has the potential to limit engagement^46^, as questions may not be asked or answered so freely. Any unwillingness to engage in open discussion may have the added effect of reducing performance if intraoperative communication is silenced or censored^42,52^.

#### Storage and security

The recommendations for OR recording security ranged from physical, under lock and key, to encryption techniques (two-way hashing mechanism), password protection, and anti-hacking or firewall software with storage media, including physical discs, software platforms, and cloud databases^38,44,47,52,60^. File names should not contain patient identifiers. For example^38^, research groups that need to associate performance analysis with surgical outcomes can use a two-way patient identifier hashing mechanism to encrypt medical record numbers and allow the association between specific patient videos and their outcomes without sharing any sensitive data with a third party^47^.

### Autonomy

Respect for patient autonomy was discussed in 10 of the included publications, including implications with regard to consent and data use (9 publications), as well as editing of data (5 publications).

#### Consent and data use

All of these publications recommended that consent is obtained from patients and three publications^47,52,60^ recommended that the surgical team also provide consent for recording. In cases where consent cannot be obtained, one study stressed that recording may not be pursued^42^, whereas two claimed that it can, provided that it is an emergency situation^55^ and/or consent is acquired before any use of the data^52^. Three publications suggested that the scope of the data, including potential uses (particularly commercial), must be discussed at the time of consent and that each potential use should be explicitly stated^42,47,52^. In a survey, a majority of interviewees felt recordings should be restricted to medical professionals. Regarding withdrawal of consent, three publications stated this should be possible at any time^52^ and should not affect patient care^42,47,52^. No direct comparison was made between the opinions of patients and physicians.

#### Editing

Much of the utility of operative videos for education, publication, and evaluation^47,52^ relies on edits, as otherwise the data would be too large and viewing would be too time-consuming^39^. However, editing and compressing an hours-long operation into a few minutes introduces bias^39,42,47^ and is likened by two authors to tampering with the medical record^52^ or hiding physician mistakes^40^. Two publications recommended that the original file be maintained^47,52^.

### Justice

The principle of the fair treatment of individuals, as well as the equitable allocation of healthcare resources, was discussed in relation to data ownership (11 publications), cost (2 publications), credentialling (5 publications), and distributing surgical knowledge (3 publications).

#### Ownership

A key ethical and legal topic discussed was that of data ownership. Eleven publications debated who owned the surgical data acquired and their recommendation of stakeholder varied greatly. Two stated that patients own their own video recording^40,55^, while one recommended that they are allowed to view the recording^52^. Four argued for its inclusion in the medical record and, as such, the ownership may fall to the institution in which it was created^44,47,52,56^, but this was considered not necessarily the case if the recording was for quality-improvement initiatives^47^. Three publications argued the opposite, stating that anonymized data should not be included in the medical record^11,38,39^ and two suggested no definitive owner^42,54^. One study argued that the surgeon may be entitled to an operative video given that the gestures of the surgery represent the culmination of the surgeon’s education and experience^38^. Two of the above recommended that it should be explicit during the consent process whose property the video recording will be^44,52^. Only one proposed shared data ownership between multiple stakeholders and raised the issue of ownership regarding potential monetization^39^.

#### Cost

In a cross-sectional survey, the cost of OR recording was raised by a minority of subjects^40^. One publication argued the opposite, stating that if recording improves performance and outcomes, then the system overall will be cost saving^36^, although no formal cost–benefit analyses were performed.

#### Credentialling/reducing bullying/discriminatory behaviours

Improved credentialling of trainees (three publications), reduced discriminatory behaviours (two publications), and better assessment of surgical skill (two publications) were proposed. Four publications suggested that OR recording materially evidences performance and can be used to objectively track a trainee’s progress, reducing bias in career advancement^21,36,44,61^. This can also be extended to senior surgeons instead of traditional surrogates (for example outcomes or volumes)^38,44^. OR recording may also reduce derogatory OR behaviour (discussed in two publications)^36,40^.

#### Distributing surgical knowledge

The use of videos to allow for more even and equitable distribution of surgical knowledge^62^ to lower-income countries was discussed, with two publications suggesting that open use of video recording allows new procedures to be better disseminated and ultimately makes surgery better^36,43,52^. One study suggested that audiovisual recording coupled with tele-mentoring would allow expert surgical advice to be given at a distance^43^.

## Discussion

Although the premise is old, understanding and advancing surgery through its observation has been recently portrayed as a new field and one which is rapidly expanding due to a new capability to aggregate and store recordings and use automated methods to analyse them^63^. Surgical videos, with their high frame number, contrast, and content, qualify as big data^64^, which can be analysed by AI^65^. It is hoped that such detailed machine analysis can provide new metrics and identify otherwise hidden patterns indicative of success and failure, giving better insight^66^. Surgery is not unique in looking to recordings to improve quality. For comparison, black box recorders were first made mandatory in the airline industry in 1960^67^ and all new cars produced within the European Union from 2022 require ‘electronic data recorders’^68^. However, whereas historically medicine and medical ethics have taken a paternalistic approach to the doctor–patient relationship, this has changed dramatically in recent decades. Prompted in part by multiple controversies surrounding instances of this relationship being abused throughout the 20th century^69–71^ and previously (for example medical schools procuring human bodies from grave robbers), greater importance is now rightly placed on patient autonomy and shared decision-making. Therefore, it is important to consider deeply how surgical recording sits ethically in the modern era of clinical practice as much as the mere technological capability.

To help with this, as in other areas, the pillars of Beauchamp and Childress can be usefully applied, weighing beneficence against the issues presented in the other pillars. While surgical training (where operative videos may help offset the lower case-volume experience of today’s trainee due to changes in working hours, advances in non-surgical management and technologies, and the COVID-19 pandemic, among other factors^72–74^), quality-improvement measures more generally (which intraoperative recordings can assist by providing a uniquely objective, visual record of events to assist investigations of how errors may have occurred or equally confirm that no error occurred and help with open disclosure, especially where mandated^75–77^), and surgical-device development (including post-marketing surveillance) are clear areas where targeted quality initiatives addressing both technical and non-technical aspects of surgery can be augmented through the use of surgical videos, such use cases cannot be simply advocated in isolation from patient, practitioner, and societal rights, responsibilities, and obligations. Non-maleficence for instance challenges beneficence if privacy concerns are disrespected and autonomy and justice also need consideration regarding recording consent and the storing, editing, and fair and equitable use of a video, allocation of resources, and ownership respectively. Until all aspects are fully considered, it is premature to conclude, as several publications do^36,39,44,47^, that implementing surgical recording falls already and automatically under a surgeon’s professional duty of care. Overall, publications in this systematic review focus more heavily on positive potential and only on the individuals immediately involved (that is patient and clinician privacy) without considering the implications of widespread operative recording (including, for instance, the idea of archival ‘hoarding’ of graphic recordings—including imagery such as patient genitalia—might seem improper or even shocking to the general public as may the selling of medical data to commercial entities and/or the use of data insights garnered from jurisdictions with different citizen rights from where they are being applied). Further, there seems to be a dichotomy between published opinions regarding privacy between patients and staff, with patients, but not staff, being concerned about their individual privacy, despite evident exposure risks. Also, importantly, all publications found by systematic search were published in the 21st century (and the majority since 2010) and also North America and Europe (84 per cent) were predominant as the origin (similar to a 2021 systematic review that found 94 per cent of 70 380 recordings were similarly originated^78^). This is possibly because the technological capability is often commercially provided and the perhaps greater potential for commercial exploitation by healthcare practitioners/providers in more technology-driven capitalist economies.

It is clear from this systematic review that data management and ownership are crucial areas for further clarification and research. Legal frameworks that require researchers to make sure that personal data collected from patients and healthcare professionals are used fairly and lawfully, for limited and specifically stated purposes, in an adequate, relevant, and considered manner, and kept safe and secure and stored for no longer than is absolutely necessary^15^ are in more common use regarding biological data and video data bring new challenges. Anonymized and sufficiently encrypted data typically do not constitute personal data as, for example, under the European Union GDPR, but the infamous Dinerstein *versus* Google case illustrates very well the risks of reidentification^79,80^, such as through data triangulation, and data-fusion and new technologies (such as facial recognition and even quantum computing) may pose severe risks for this in the near future. Traditionally, consent for data recording should be able to be withdrawn at any stage, but more sophisticated analytic methods make processed data intrinsic to the methods, making complete removal of such data where the data has already been used (for example in algorithmic training sets) difficult if not impossible.

Data ownership is a much debated concept and term, with many different aspects, ranging from ethical aspects, enshrined in the principles of autonomy, to overlapping legal aspects, ranging from privacy protection and personal rights to intellectual-property rights^80^. Interestingly, privacy laws in Europe already include the caveat that data collected for healthcare quality improvement may not be required to be added to a patient’s medical record (if a video is made part of the medical record then access is often grantable under Freedom of Information Acts^81,82^). If the same holds true for anonymized surgical recordings, a question arises over legal ownership and potential ‘secondary’-use restrictions. This is particularly important when the potential commercial use of these data is considered *versus* other important outputs that need weighing, such as public interest and societal good through better surgery. As the commercial value of ‘big data’ becomes apparent to the general population, controversies have arisen where medical data generated for research purposes were subsequently utilized for commercial purposes without the consent of the participants^83,84^. Certainly the idea of possession (whether surgeon, patient, or hospital) ascribing ownership should not be automatically assumed. Another question that arises is whether patients should be allowed to view or hold a copy of their recording? There are many possible reasons why, alongside garnering evidence for potential medical malpractice, including increased transparency in ORs, understanding procedures and practices, and even patients’ or their loved ones’ recovery from the emotional trauma of surgery^56^. Patient ownership of such material naturally confers similar responsibilities (including in such matters as safe storage and restrictions regarding public sharing), as surgical videos may contain data belonging to others (in contrast to the standard medical record, which only contains information specific to the patient). There are also concerns regarding harm or the risk of misinterpretation in viewing surgical videos, especially perhaps when the outcome is known given that the standard is reasonable competence and not perfection and no precise definition as yet exists as to what represents error *versus* acceptable variation. To this end, some publications promote a shared ownership model (potentially analogous to a biobank) and/or a catalogued library of the operation, which may prove sufficient for many of the reasons operations may need to be viewed.

There are a number of limitations to this systematic review, mostly relating to the quality of the publications, as discussed above. While societal cost^36,40^ and knowledge distribution^36,43,52^ were discussed, overall, there was a paucity of literature pertaining to surgical video recording and the general population. Therefore, it was not possible to obtain an adequate estimation of the impact of the ethical implications of OR recording on the general population and society. Ethical considerations are not as amenable to the standard systematic review models predicated on measurable interventions, outcomes, and evidence, and, too often, there is overlap with legal aspects. Nonetheless, this systematic review generally serves the purpose of showing the gaps present in the current literature.

Surgical video recording is a growing reality with great potential, but which also presents ethical concerns. Certain general principles are already clear, yet to make this capability truly beneficial and fully operational, many issues still need to be addressed. With clarity, sincerity of purpose, and a responsible, balanced, proportional approach that takes into account practical realities, the value of this resource can be sustainably realized. While jurisdictions may differ, we as a surgical, scientific, and professional community have a responsibility, not only to apply existing ethical and legal frameworks, but to develop broadly applicable and workable guidelines. Given the complexity of the issues, and noting that many relevant aspects, such as liability and bias, still have to be covered, this will require close collaboration of interdisciplinary and inclusive teams, regulators, and society.

## Supplementary Material

zrad063_Supplementary_Data

## Data Availability

All data generated or analysed during this study are included in this published article (and its *Supplementary material*).
