# Supplementary material for: Ethical perspectives on surgical video recording for patients, surgeons and society: systematic review
Source: BJS Open. 2023 Jun 24;7(3):zrad063. doi: 10.1093/bjsopen/zrad063 (PMC10290491; doi:10.1093/bjsopen/zrad063)
Supplement: zrad063_Supplementary_Data [file zrad063_supplementary_data.zip › Supplementary_Material.docx]

**Ethical Perspectives on Surgical Video Recording for Patients, Surgeons and Society: Systematic Review with Narrative Synthesis**

Ross Walsh^1^, Emma Kearns^2^, Alice Moynihan^2^, Sara Gerke^3^, Mindy Duffourc^3,4^,

Timo Minssen^5^, Ronan A Cahill^1,2^

^1^Department of Surgery, Mater Misericordiae University Hospital, Dublin, Ireland

^2^UCD Centre of Precision Surgery, University College Dublin, Ireland.

^3^Penn State Dickinson Law, Carlisle, PA, USA.

^4^New York University Law School, New York, NY, USA.

^5^Centre for Advanced Studies in Biomedical Innovation Law (CeBIL), University of Copenhagen, Denmark

**Corresponding author.** Prof Ronan Cahill, 47 Eccles Street, Dublin 7, Ireland. Email: [ronan.cahill@ucd.ie](mailto:ronan.cahill@ucd.ie) Telephone: 00353 1 716 4597

**Supplementary Materials - Index**

| **Supplementary Methods** |  |
| --- | --- |
| Table S1. Complete Search Strategy | *pag. 2* |
| **Supplementary Figures and Tables** |  |
| Table S2. Categorised List and Brief Summary of Publications in each of the four pillars of medical ethics. | *pag. 4* |
| **References** | *pag. 12* |

**Supplementary Methods**

**Table S1.** Complete Search Strategy used for the performance of this systematic review

*PubMed*

("operating rooms"[MeSH Terms] OR "surgery"[Title/Abstract] OR "surgeons"[Title/Abstract] OR "surgical procedures, operative"[MeSH Terms])

AND ("video recording"[MeSH Terms] OR "video recording"[Title/Abstract] OR "black box"[Title/Abstract] OR "black box recording"[Text Word] OR "digital recording"[Text Word])

AND ("ethics"[Text Word] OR "consent"[Title/Abstract] OR "Informed consent"[Text Word] OR "confidentiality"[Text Word] OR "privacy"[Text Word])

N = 273

*Embase*

| No. | Query | Results |
| --- | --- | --- |
| #19 | #6 AND #11 AND #18 | **779** |
| #18 | #12 OR #13 OR #14 OR #15 OR #16 OR #17 | **448393** |
| #17 | 'ownership':ti,ab,kw | **20444** |
| #16 | 'privacy':ti,ab,kw | **23715** |
| #15 | 'confidentiality':ti,ab,kw | **16044** |
| #14 | 'informed consent':ti,ab,kw | **92106** |
| #13 | consent:ti,ab,kw | **152984** |
| #12 | 'ethics' | **277052** |
| #11 | #7 OR #8 OR #9 OR #10 | **110284** |
| #10 | 'digital recording':ti,ab,kw | **442** |
| #9 | 'black box recording':ti,ab,kw | **6** |
| #8 | 'black box':ti,ab,kw | **4915** |
| #7 | 'videorecording'/exp OR 'videorecording' | **105046** |
| #6 | #1 OR #2 OR #3 OR #4 OR #5 | **2314114** |
| #5 | 'surgical procedures':ti,ab,kw | **94837** |
| #4 | operative:ti,ab,kw | **470060** |
| #3 | surgeon:ti,ab,kw | **164689** |
| #2 | surgery:ti,ab,kw | **1920105** |
| #1 | 'operating room'/exp OR 'operating room' | **74338** |

**Supplementary Figures and Tables**

**Table S2:** Categorised List and Brief Summary of Publications in each of the four pillars of medical ethics. ORBB: operating room black box; QI: quality improvement.

| **Beneficence** | Education | \| Gallant \| Interviewees felt OR recordings could improve teaching \| \| --- \| --- \| \| Gabrielli \| Video based coaching / Surgeon and staff \| \| Darrow \| Improve intra-op teaching with tele-mentoring with Google Glass \| \| Doyen \| Allows trainees to learn from mistakes by re-watching operative recordings giving more focused training \| \| Thia \| Video based coaching giving more focused training \| \| Hung \| Tele-mentoring can shorten the learning curve for laparoscopic surgery \| \| Langerman \| May limit teaching if surgeon unwilling to engage in open discussion if recorded \| \| Chang \| Allow juniors to view surgery from operator’s point of view \| \| Evans \| Surgical videos can enhance education and training \| \| Turnbull \| Increase trainee learning of cases and allow reflective practice by looking back on surgeries \| \| Xiao \| Surgical education with targeted feedback and OR setup dynamic education \| |
| --- | --- | --- | --- | --- | --- | --- | --- | --- | --- | --- | --- | --- | --- | --- | --- | --- | --- | --- | --- | --- | --- | --- | --- | --- |
|  | Performance | \| Cahill \| Retrospective case review without the constraints of memory. Improve technical skill by watching back own movements. Allows for life-long learning. \| \| --- \| --- \| \| Gallant \| Majority of the 49 interviewees believed recording would affect performance (Hawthorne effect) \| \| Jue \| Accurately record the details of the procedure allowing for more accurate retrospective case review improve technical performance/skill \| \| Gabrielli \| Video can capture team performance, document technical skills and can be powerful tools to analyse system factors. May however reduce talking making for a more stressful work environment and reducing performance \| \| Thia \| It can allow self-assessment and evaluation of operations and in ophthalmology numerous self-assessment tools have been developed. It can also improve performance due to increased accountability or the Hawthorne Effect \| \| Langerman \| Black box concept would help study OR dynamics and interactions among team members. This data would be beneficial in testing solutions and new technologies to improve communication and operating room performance \| \| Prigoff \| Video recording can improve operative technique. Recording can support improved attentiveness and meticulousness leading to improved surgical outcomes \| \| Chang \| The glasses had the potential to improve communication performance with augmented reality recordings and allow participation of senior colleagues in surgical decision making without being physically present \| \| O'Mahoney \| Video can improve surgical skill and encourage standardization in surgical process. Hawthorne effect may improve performance \| \| Grenda \| Retrospective case review to improve skill and technique \| \| Bonrath \| Video enhanced coaching resulted in increased skill acquisition and less intra-operative errors \| \| Turnbull \| Receiving real-time feedback from video recordings has the long-term benefit of improving surgical performance and thus patient safety and outcomes. May affect surgeon in different ways however \| \| Xiao \| Video can capture performance and improve communication intra-operatively \| |
|  | Transparency | \| Jesudason \| Clear objective record of what happened and can help patients to understand what went wrong \| \| --- \| --- \| \| Gallant \| 100% of interviewees saw benefit in having an objective record of an operation. Many also believed that increased transparency could improve patient understanding \| \| Thia \| Video recording improves documentation and can be used for quality improvement and audit \| \| Langerman \| Serves as an objective record. Recordings of the entire OR allow an evaluation of unrecognized risks and hazards and provide more effective root cause analysis and peer review after adverse event. Can aid patient understanding and allow them to make more informed health decisions \| \| Prigoff \| Video recording provides a more objective and reliable account of the operation than self-reported dictation \| \| O'Mahoney \| Routine surgical recording would avoid incomplete operative notes and assist quality improvement efforts \| \| Kocyildirim \| Video recording of paediatric cardiac surgery appeared to play an important role in helping parents come to terms with the emotional trauma of the surgery \| |
|  | Artificial Intelligence | \| Jesudason \| Video recording can be used to train AI to look for patterns that might be associated with success and failure, and to identify surgeons whose strengths or weaknesses can be respectively emulated or addressed \| \| --- \| --- \| \| Cahill \| New insight into surgery, benchmarking quality improvement initiatives, guiding device and decision-support system development and allow reformatting or simplification of operative technique \| \| Thia \| AI can use motion analysis to examine and improve performance \| |
|  | Quality Improvement | \| Cahill \| Analysis of recording can aid quality improvement initiatives \| \| --- \| --- \| \| Jue \| Video recording can be used for error detection, thus serving the overall purpose of improving the quality of care \| \| Gabrielli \| Operative video can allow analysis of human and system factors to improve quality \| \| Doyen \| ORBB can be used for error detection to investigate but human and system factors. Examples include radiation safety behaviours, WHO checklist and OR efficiency \| \| Thia \| Video can be used for QI initiatives and documentation/audit \| \| Langerman \| Recordings of the entire OR allow an objective evaluation of unrecognized risks and hazards and provide more effective root cause analysis and peer review after adverse event and quality improvement \| \| O'Mahoney \| Can be used to monitor intraoperative technique or ensure that key surgical steps are performed for quality improvement. Objective surgical recording would assist quality improvement efforts \| \| Bonrath \| Error detection to drive quality improvement \| \| Couat \| Video-based observational and analytical approach to improve OR safety, identify incidents and learn from them \| \| Xiao \| Video analysis can compare events to best practice templates to learn from mistakes ad aid quality improvement \| |
| **Non- Maleficence** | Confidentiality | \| Jesudason \| Confidentiality of patient identification should be considered as should the personal privacy of surgeons and staff \| \| --- \| --- \| \| Gordon \| 45% of respondents concerned about data security from ORBB \| \| Filicori \| GDPR/HIPAA guidelines should be followed. Data should be stored for the shortest time possible and anonymized as early as possible. Audio recording can give important information but risk identifying patients \| \| Gallant \| Majority of interviewees had concerns whether they would be recognizable particularly in surgeries relating to face or genitals \| \| Gabrielli \| With recording of operations there is a risk that patient or physician identity may be exposed \| \| Darrow \| Quantitatively characterized the risk of potential privacy exposure as 0.4 exposures/min in OR \| \| Van Dalen \| Data should be handled in line with GDPR or HIPAA. Individuals should be strictly and clearly informed about what happens to their personal data. Data obtained should be anonymised as soon as possible \| \| Thia \| Gives recommendations about maintaining confidentiality. Video recording should start only when procedure is ready to start. Blurring should be used if unable to avoid identifiable structure and part of recordings not relevant to procedure should be removed. Audio could be omitted and add a verbal commentary postoperatively if needed. Care should be taken with naming of such files \| \| Prigoff \| Video and audio recordings risk breaching privacy of both patient and team and given the large potential audience may cause distress if either are identifiable \| \| Chang \| Google Glass automatically backs up onto Google cloud technology which leads to the transferal of potentially private and protected information onto unprotected servers. WIFI could be disabled and only data uploaded to secure local network to avoid recording patient identifiers \| \| O'Mahoney \| Protocols for video recording would reduce privacy loss. At present it is common that patient photographs and video over nonencrypted cellular and email systems, this integration into a formal data collection and storage system would reduce this patient privacy loss \| \| Silas \| Post processing of data can ensure anonymity \| \| Turnbull \| Privacy of patient and team at risk. Every member of the OR should be aware of the recording, particularly if audio recording is taking place \| \| Henken \| Privacy of personal data requires that patients and professionals be informed about the purpose and the nature of video recording and that consent be obtained whenever possible \| \| Xiao \| Identifying information about the patient should be removed \| |
|  | Open Discussion | \| Gabrielli \| Open discussion in the OR creates a better environment but the video recording may prevent this \| \| --- \| --- \| \| Langerman \| Recording might limit teaching in the OR as surgeons may be unwilling to engage in open discussion \| \| Turnbull \| Video recording process may serve as a distraction or provoke anxiety and prevent effective communication. This may affect performance \| |
|  | Security | \| Filicori \| Videos should be stored securely, either in a physically locked area or in password encrypted files \| \| --- \| --- \| \| Thia \| Secure systems should be in place at institutions to ensure safe storage of video recordings. Digital security methods such as encryption, password protection, and antihacking software should be used. File names should not include any patient identifiers such as the patient's name, date of birth, or medical record number. Consideration should be made about automatic device uploading to cloud data bases \| \| Prigoff \| Most hospitals have very strict guidelines regarding patient records and any identifiable information. However, these videos are routinely stored on personal devices and used in conferences and classrooms where the security is less stringent \| \| Turnbull \| There is a risk that video recording may violate the privacy of patients, even when consent has been obtained, if the material is subsequently misused. Recordings must receive the same care and protection as any other medical record. If videos are being stored, they must be anonymized with the removal of any patient identifiable data. Storage must be secure, with measures taken to prevent or mitigate the consequences of theft \| |
| **Autonomy** | Consent | \| Jesudason \| Consent should be sought for making and sharing video recordings \| \| --- \| --- \| \| Filicori \| Consent often doesn’t cover scope of potential uses of video. Any commercial use of video data should be disclosed to the patient and explicit consent obtained and documented \| \| Gallant \| Majority of subjects believed that consent should be obtained. They also felt recordings should be restricted to medical professionals. Many did not want videos to be uploaded to internet \| \| Gabrielli \| Consent should be explicitly obtained. Videos should be deleted if consent is withdrawn. Filming should only be done is consent can be obtained. Uncertainty of how video recording may be used in future \| \| Van Dalen \| Consent should include informing patients strictly and clearly informed about what happens to their personal data \| \| Thia \| Explicit consent should be obtained and the discussion should include patient confidentiality and anonymity, the purpose of video recording, ownership and storage of video recording \| \| Prigoff \| Informed consent must be obtained. Before recording is performed, the patient must understand the purpose for the recording, its audience, and how long the recording will be kept, as the recording may only be used for the purposes for which the patient's consent has been obtained. The patient must be assured that refusal to consent to a recording will not affect their quality of care. The team must also give consent for recording or have the option to opt out \| \| Turnbull \| Patient consent needs to be reliably and appropriately sought. If any doubt exists, the simplest options are either to seek consent or to decide against pursuing a video record in the first place \| \| Xiao \| Consent should be sought but may be waived in emergency procedures. \| |
|  | Editing | \| Cahill \| Hours long operation compressed into a few minutes understates complexity and subject to bias however unedited presentation is too time consuming \| \| --- \| --- \| \| Gallant \| Interviewees expressed concern over the use of data editing of video to hide mistakes of medical professionals \| \| Gabrielli \| Questions whether video should be freely edited or erased \| \| Prigoff \| Video editing is beneficial for education and evaluation but inherently brings bias. Changes should be documented and original recording should be kept \| \| Turnbull \| Debate exists as to whether surgeons should be allowed to edit their surgical videos Benefit for presentation or publication but may be considered tampering with medical record. Recommend that original file be maintained \| |
| **Justice** | Ownership | \| Filicori \| Surgical video could be used to generate commercial products. The gestures/motion of the surgery represent the culmination of a surgeon’s education and experience. Data from a video recording used for quality improvement, education, or research should not be included in the medical record \| \| --- \| --- \| \| Cahill \| Outdated ownership models could lead to misuse or exclusion from use of data. The act of recording and/or camera ownership or data storage does not confer sole proprietorship. Surgical video relating to a patient's ‘innards’ does in itself comprise personal data either of the patient or the surgeon as neither are ‘recognisable’ from the images alone. Shared data ownership to benefit all is proposed \| \| Gallant \| Ownership was rarely brought up by respondents but when prompted 88% felt they own the recording as it is their body. No further justification for this was made. \| \| Gabrielli \| Ownership of data is questioned. Stakeholders of the patient, the physician, the subject and the researcher are identified \| \| Van Dalen \| Only identifying information should be a part of the medical record. This data must be accessible to the patient and stored for at least 5 years \| \| Thia \| Ownership should be clarified as part of the consent process. A video recording is generally considered to be the property of the institution in which it was created. \| \| Prigoff \| Should be part of the medical record as it a recorded event involving the patient, that can be used later for patient care. This is not the case if it is designated as quality improvement however. This calls into question whether the patient, physician, or hospital owns the recording \| \| Turnbull \| The surgeon who created the video does not own it. Medical record of any type legally belongs to the employing body and depending on the law the patient may own to video or be entitled to see it. It should be made explicit during the consent process whose property the video recording will be. Surgeons should ensure that they understand who owns the copyright and intellectual property rights \| \| Henken \| Unclear on whether should be included in medical record. Anything relevant to providing good care should be included, but the care providers decide on the exact content of the patient record \| \| Xiao \| Patients should have the option of viewing their own recordings and to destroy them if they want \| \| Kocyildirim \| VR considered part of the medical record \| |
|  | Cost | \| Jesudason \| May actually be a good allocation of resources and by improving healthcare outcomes resulting in cost savings for institutions \| \| --- \| --- \| \| Gallant \| A minority of subjects questioned the cost of ORR \| |
|  | Fairness | \| Jesudason \| Video recording provides material evidence of skill. This can reduce discrimination and bias in training scheme interviews and promotion applications. It may also reduce discriminatory behaviours during surgery. \| \| --- \| --- \| \| Filicori \| Video can be used to assess performance and surgical skills. Without clear, standardized metrics upon which to judge surgical technique, hospital administrators rely on indirect proxies, such as outcomes or case volume, that may not be accurate. While some surgeons may be concerned about unwarranted scrutiny into their operative techniques, this push towards objective metrics is an evolution that mirrors the shift to evidence-based medical practice. \| \| Gallant \| 30% of interviewees believed that OR recording would reduce derogatory behaviour in the OR \| \| Doyen \| ORBB facilitates objective tracking of trainees’ progress throughout training, thereby stimulating competency-based education \| \| Thia \| Video based assessment reduces bias \| |
|  | Distribution | \| Jesudason \| Video recording can allow for surgical knowledge to be easily distributed to low income countries. This can make surgery more affordable \| \| --- \| --- \| \| Darrow \| Tele-mentoring allows expert surgical advice to be given anywhere in the world \| \| Turnbull \| New procedures and techniques can be disseminated through surgical video \| |

**References**

1. Bishop WJWJ. The Early History of Surgery. London: Robert Hale Ltd; 1960. 231- p.

2. Ellis H, Abdalla S. A history of surgery: CRC Press; 2018.

3. Schofield JC. Principles of aseptic technique. Essentials for Animals Research; A Primer for Research Personnel. 1994:59-77.

4. Durkin C, Vaghadia H, Price J. Video teaching in the operating room. Canadian Journal of Anesthesia. 2012;59.

5. Mascagni P, Padoy N. OR black box and surgical control tower: Recording and streaming data and analytics to improve surgical care. J Visc Surg. 2021;158(3s):S18-s25.

6. Jung JJ, Jüni P, Lebovic G, Grantcharov T. First-year Analysis of the Operating Room Black Box Study. Ann Surg. 2020;271(1):122-7.

7. Cartucho J, Tukra S, Li Y, S. Elson D, Giannarou S. VisionBlender: a tool to efficiently generate computer vision datasets for robotic surgery. Computer Methods in Biomechanics and Biomedical Engineering: Imaging and Visualization. 2021;9(4):331-8.

8. Fisher R, McDermott K, Nair R, Davies B, Christodoulou A, Cardiff E, et al. Integration of a virtual surgeon collaborative augmented reality platform into robotic surgery: An IDEAL framework stage 1 study. European Urology Open Science. 2020;19:e1321.

9. Eitel DR, Yankowitz J, Ely JW. Legal implications of birth videos. J Fam Pract. 1998;46(3):251-6.

10. Van Dalen ASHM, Legemaate J, Schijven MP. Video and medical data recording in the operating room; The current legal framework outlined. Surgical Endoscopy and Other Interventional Techniques. 2017;31(2):S56.

11. van Dalen A, Legemaate J, Schlack WS, Legemate DA, Schijven MP. Legal perspectives on black box recording devices in the operating environment. Br J Surg. 2019;106(11):1433-41.

12. Blaauw CB, van den Dobbelsteen JJ, Hubben JH. [Legal aspects of video registration during operations--the digital operating room assistant: opportunity or threat?]. Ned Tijdschr Geneeskd. 2011;155(32):A3487.

13. Cumpanas AA, Ferician OC, Latcu SC, Pricop C, Bardan RT. Ethical, legal and clinical aspects of live surgery in urology - Contemporary issues and a glimpse of the future. Wideochirurgia I Inne Techniki Maloinwazyjne. 2017;12(1):1-6.

14. O'Sullivan S, Nevejans N, Allen C, Blyth A, Leonard S, Pagallo U, et al. Legal, regulatory, and ethical frameworks for development of standards in artificial intelligence (AI) and autonomous robotic surgery. Int J Med Robot. 2019;15(1):e1968.

15. General Data Protection Regulation (GDPR), (2018).

16. Health Insurance Portability and Accountability Act. , (2000).

17. Childress TLBaJF. Principles of Biomedical Ethics: Oxford University Press; 2019.

18. Druedahl LC, Lebret A, Minssen T. ELSI Implications of Prioritizing Biological Therapies in Times of COVID-19. J Law Med Ethics. 2020;48(3):579-82.

19. Ross Walsh EK. A Systematic Review of the Ethical Perspectives on Surgical Video. 2022.

20. Doyen B, Gordon L, Soenens G, Bacher K, Vlerick P, Vermassen F, et al. Introduction of a surgical Black Box system in a hybrid angiosuite: Challenges and opportunities. Phys Med. 2020;76:77-84.

21. Chang JYC, Tsui LY, Yeung KSK, Yip SWY, Leung GKK. Surgical Vision: Google Glass and Surgery. Surgical Innovation. 2016;23(4):422-6.

22. Williams JB, Mathews R, D'Amico TA. "Reality surgery" a research ethics perspective on the live broadcast of surgical procedures. Journal of Surgical Education. 2011;68(1):58-61.

23. Smith A. Urological live surgery - an anathema. BJU Int. 2012;110(3):299-300.

24. Rao AR, Karim O. A benedictory ode to urological live surgery. BJU Int. 2013;112(1):11-2.

25. Salami S, Elsamra S, Friedlander J, George A, Duty B, Okeke Z, et al. Perception of urologists performing live case demonstration (LCD)-To be or not to be? Journal of Endourology. 2013;27:A55.

26. Philip-Watson J, Khan SAA, Hadjipavlou M, Rane A, Knoll T. Live surgery at conferences - Clinical benefits and ethical dilemmas. Arab Journal of Urology. 2014.

27. Antonelli A, Carrieri G, Porreca A, Veneziano D, Artibani W. Live Surgery: Is Operating at Home the Way Forward? Eur Urol. 2018;74(4):403-4.

28. Liverneaux P. Should we ban Live Surgery? J Visc Surg. 2019;156(4):279-80.

29. Min SK. Ethics of live surgery demonstration or broadcast: Is it beneficial to the patients? Vascular Specialist International. 2020;36(1):4-6.

30. Choi PJ, Oskouian RJ, Tubbs RS. Telesurgery: Past, Present, and Future. Cureus. 2018;10(5):e2716.

31. Evans CH, Schenarts KD. Evolving Educational Techniques in Surgical Training. Surgical Clinics of North America. 2016;96(1):71-88.

32. Medicine CoEB. Levels of Evidence. In: Medicine CfE-B, editor.

33. Popay J. Guidance on the Conduct of Narrative Synthesis in Systematic Reviews: Product from the ESRC Methods Programme. 2006.

34. Campbell M, McKenzie JE, Sowden A, Katikireddi SV, Brennan SE, Ellis S, et al. Synthesis without meta-analysis (SWiM) in systematic reviews: reporting guideline. BMJ. 2020;368:l6890.

35. Gallant JN, Brelsford K, Sharma S, Grantcharov T, Langerman A. Patient Perceptions of Audio and Video Recording in the Operating Room. Annals of surgery. 2021.

36. Li B, Wang Y, Al-Jarallah O, Hoogenes J, Matsumoto ED. Perceptions and attitudes of learners towards video recording of their operative performance for assessment of surgical skills. Journal of Urology. 2021;206(SUPPL 3):e189-e90.

37. Gabrielli M, Valera L, Barrientos M. Audio and panoramic video recording in the operating room: legal and ethical perspectives. J Med Ethics. 2020.

38. Darrow DP, Spano A, Grande A. The Potential for Undue Patient Exposure during the Use of Telementoring Technology. Cureus. 2020;12(4):e7594.

39. Thia BC, Wong NJ, Sheth SJ. Video recording in ophthalmic surgery. Surv Ophthalmol. 2019;64(4):570-8.

40. Hung AJ, Chen J, Shah A, Gill IS. Telementoring and Telesurgery for Minimally Invasive Procedures. Journal of Urology. 2018;199(2):355-69.

41. Langerman A, Grantcharov TP. Are We Ready for Our Close-up? Annals of Surgery. 2017;266(6):934-6.

42. Turnbull AMJ, Emsley ES. Video recording of ophthalmic surgery-ethical and legal considerations. Survey of Ophthalmology. 2014;59(5):553-8.

43. Xiao Y, Schimpff S, Mackenzie C, Merrell R, Entin E, Voigt R, et al. Video technology to advance safety in the operating room and perioperative environment. Surg Innov. 2007;14(1):52-61.

44. Grenda TR, Pradarelli JC, Dimick JB. Using Surgical Video to Improve Technique and Skill. Ann Surg. 2016;264(1):32-3.

45. Jesudason E. Surgery should be routinely videoed. J Med Ethics. 2022.

46. Prigoff JG, Sherwin M, Divino CM. Ethical Recommendations for Video Recording in the Operating Room. Ann Surg. 2016;264(1):34-5.

47. O'Mahoney PR, Yeo HL, Lange MM, Milsom JW. Driving Surgical Quality Using Operative Video. Surg Innov. 2016;23(4):337-40.

48. Kocyildirim E, Franck LS, Elliott MJ. Intra-operative imaging in paediatric cardiac surgery: the reactions of parents who requested and watched a video of the surgery performed on their child. Cardiol Young. 2007;17(4):407-13.

49. Wauben LS, van Grevenstein WM, Goossens RH, van der Meulen FH, Lange JF. Operative notes do not reflect reality in laparoscopic cholecystectomy. Br J Surg. 2011;98(10):1431-6.

50. Council IM. Eight Domains of Good Professional Practice 2022 [Available from: <https://www.rcpi.ie/professional-competence/information-for-enrolled-doctors/domains-of-good-professional-pracatice-01/>.

51. Cahill RA, Mac Aonghusa P, Mortensen N. The age of surgical operative video big data - My bicycle or our park? Surgeon. 2022;20(3):e7-e12.

52. Jue J, Shah NA, Mackey TK. An Interdisciplinary Review of Surgical Data Recording Technology Features and Legal Considerations. Surg Innov. 2020;27(2):220-8.

53. Bonrath EM, Dedy NJ, Gordon LE, Grantcharov TP. Comprehensive Surgical Coaching Enhances Surgical Skill in the Operating Room: A Randomized Controlled Trial. Ann Surg. 2015;262(2):205-12.

54. Couat JF, Cegarra J, Rodsphon T, Geeraerts T, Lelardeux C, Sol JC, et al. A prospective video-based observational and analytical approach to evaluate management during brain tumour surgery at a university hospital. Neurochirurgie. 2013;59(4-5):142-8.

55. Gordon L, Reed C, Sorensen JL, Schulthess P, Strandbygaard J, McLoone M, et al. Perceptions of safety culture and recording in the operating room: understanding barriers to video data capture. Surg Endosc. 2022;36(6):3789-97.

56. Filicori F, Addison P. Intellectual property and data ownership in the age of video recording in the operating room. Surgical Endoscopy. 2021.

57. Silas MR, Grassia P, Langerman A. Video recording of the operating room--is anonymity possible? J Surg Res. 2015;197(2):272-6.

58. Henken KR, Jansen FW, Klein J, Stassen LP, Dankelman J, van den Dobbelsteen JJ. Implications of the law on video recording in clinical practice. Surg Endosc. 2012;26(10):2909-16.

59. Godfrey M, Walle KV, Rosser AA, Quamme SP, Greenberg C, Greenberg JA, et al. Overcoming Hurdles to Video Recording in the Operating Room for Surgical Education. Journal of the American College of Surgeons. 2019;229(4):e190.

60. Addison P, Yoo A, Duarte-Ramos J, Addy J, Dechario S, Husk G, et al. Correlation between operative time and crowd-sourced skills assessment for robotic bariatric surgery. Surg Endosc. 2021;35(9):5303-9.

61. Cookson R, Dolan P. Principles of justice in health care rationing. Journal of Medical Ethics. 2000;26(5):323-9.

62. Ieong E, Mahapatra P, Duncan J, Sadri A. Train hard, go pro-use of personalised video training in orthopaedic surgery. International Journal of Surgery. 2014;12:S84.

63. Donoho D. 50 years of data science. Journal of Computational and Graphical Statistics. 2017;26(4):745-66.

64. Challen R, Denny J, Pitt M, Gompels L, Edwards T, Tsaneva-Atanasova K. Artificial intelligence, bias and clinical safety. BMJ Qual Saf. 2019;28(3):231-7.

65. McKinsey. Big data: The next frontier for innovation, competition, and productivity 2022 [Available from: <https://www.mckinsey.com/business-functions/mckinsey-digital/our-insights/big-data-the-next-frontier-for-innovation>.

66. Brajcich BC, Stulberg JJ, Palis BE, Chung JW, Huang R, Nelson H, et al. Association Between Surgical Technical Skill and Long-term Survival for Colon Cancer. JAMA Oncol. 2021;7(1):127-9.

67. Levin M, McKechnie T, Kruse CC, Aldrich K, Grantcharov TP, Langerman A. Surgical data recording in the operating room: A systematic review of modalities and metrics. British Journal of Surgery. 2021;108(6):613-21.

68. Humm G, Harries RL, Stoyanov D, Lovat LB. Supporting laparoscopic general surgery training with digital technology: The United Kingdom and Ireland paradigm. BMC Surg. 2021;21(1):123.

69. Mattar SG, Alseidi AA, Jones DB, Jeyarajah DR, Swanstrom LL, Aye RW, et al. General surgery residency inadequately prepares trainees for fellowship: results of a survey of fellowship program directors. Ann Surg. 2013;258(3):440-9.

70. Awan M, Zagales I, McKenney M, Kinslow K, Elkbuli A. ACGME 2011 Duty Hours Restrictions and Their Effects on Surgical Residency Training and Patients Outcomes: A Systematic Review. J Surg Educ. 2021;78(6):e35-e46.

71. Fonseca AL, Reddy V, Longo WE, Gusberg RJ. Graduating general surgery resident operative confidence: perspective from a national survey. J Surg Res. 2014;190(2):419-28.

72. Kearse LE, Zeineddin A, Schmiederer IS, Korndorffer JR, Jr., Lau JN. A 20-year review of surgical training case logs: Is general surgery still general? Surgery. 2021;170(5):1347-52.

73. Humm G, Mohan H, Fleming C, Harries R, Wood C, Dawas K, et al. The impact of virtual reality simulation training on operative performance in laparoscopic cholecystectomy: meta-analysis of randomized clinical trials. BJS Open. 2022;6(4).

74. Cragg J, Mushtaq F, Lal N, Garnham A, Hallissey M, Graham T, et al. Surgical cognitive simulation improves real-world surgical performance: randomized study. BJS Open. 2021;5(3).

75. Schloendorff v. Society of New York Hospital. 105 NE 92, 93 (NY 1914): Court of Appeals of New York; 1914.

76. S.C JMHC. The Lourdes hospital inquiry: An inquiry into

peripartum hysterectomy at Our Lady of Lourdes

Hospital, Drogheda.; 2006.

77. James G. Report of the Independent Inquiry into the Issues raised by Paterson. 2020. Contract No.: Ref: ISBN 978-1-5286-1728-4, HC 31 2020-21.

78. Barry MJ, Edgman-Levitan S. Shared decision making--pinnacle of patient-centered care. N Engl J Med. 2012;366(9):780-1.

79. Health Do, Scally G. Scoping Inquiry into the CervicalCheck Screening Programme: final report. Department of Health (DoH); 2018.

80. Information H, Authority Q, Inspectorate SS. Investigation into the safety, quality and standards of services provided by the Health Service Executive to patients, including pregnant women, at risk of clinical deterioration, including those provided in University Hospital Galway, and as reflected in the care and treatment provided to Savita Halappanavar executive summary and recommendations. Health Information and Quality Authority (HIQA), Social Services Inspectorate (SSI); 2013.

81. Group DSaT. BLACK BOX FLIGHT RECORDER 2022 [Available from: <https://www.dst.defence.gov.au/innovation/black-box-flight-recorder#:~:text=Dr%20David%20Warren%20of%20Aeronautical,box%20flight%20recorder%20in%201953>.

82. Regulation (EU) 2019/2144 of the European Parliament and of the Council of 27 November 2019 on type-approval requirements for motor vehicles and their trailers, and systems, components and separate technical units intended for such vehicles, as regards their general safety and the protection of vehicle occupants and vulnerable road users. 2019/2144. European Union2019.

83. Orwell G. Nineteen eighty-four : a novel / by George Orwell. Harmondsworth, Eng: Penguin in association with Secker & Warburg; 1954.

84. Gerke S, Yeung S, Cohen IG. Ethical and Legal Aspects of Ambient Intelligence in Hospitals. JAMA. 2020;323(7):601-2.

85. Gunnarsdóttir HD CI, Minssen T, Gerke S. The Ethics and Laws of Medical Big Data. In: The Cambridge Handbook of Information Technology, Life Sciences and Human Rights. Cambridge: Cambridge University Press; 2022. (Cambridge Law Handbooks); 2022.

86. Moore v. Regents of the University of California. . 1990.

87. Henrietta Lacks: science must right a historical wrong. Nature. 2020;585(7823):7.

88. Nally DM, Kearns EC, Cahill RA. Public patient involvement and engagement via ‘think-in’ for digital cancer surgery. British Journal of Surgery. 2021;109(1):e1-e2.

89. Freedom of Information Act 2014, Oireachtas na hEireann(2014).

90. Goverment U. Freedom of Information Act 2000. 2000.
